# Detection and severity stratification of chronic liver disease using magnetic resonance intravoxel incoherent motion and elastography

**DOI:** 10.1093/radadv/umaf039

**Published:** 2025-11-14

**Authors:** Damiano Catucci, Sandro Urs von Daeniken, Verena Carola Obmann, Annalisa Berzigotti, Lukas Ebner, Johannes Thomas Heverhagen, Andreas Christe, Peter Vermathen, Adrian Thomas Huber

**Affiliations:** Department of Diagnostic, Interventional and Pediatric Radiology, Inselspital, University Hospital Bern, Bern, 3010, Switzerland; Graduate School for Health Sciences, University of Bern, Bern, 3012, Switzerland; Department of Diagnostic, Interventional and Pediatric Radiology, Inselspital, University Hospital Bern, Bern, 3010, Switzerland; Department of Diagnostic, Interventional and Pediatric Radiology, Inselspital, University Hospital Bern, Bern, 3010, Switzerland; Department of Radiology, Zuger Kantonsspital, Baar, 6340, Switzerland; Division of Hepatology, Department of Visceral Surgery and Medicine, Inselspital, University Hospital Bern, Bern, 3010, Switzerland; Department of Diagnostic, Interventional and Pediatric Radiology, Inselspital, University Hospital Bern, Bern, 3010, Switzerland; Department of Radiology and Nuclear Medicine, Lucerne Cantonal Hospital, University of Lucerne, Lucerne, 6000, Switzerland; Department of Diagnostic, Interventional and Pediatric Radiology, Inselspital, University Hospital Bern, Bern, 3010, Switzerland; Department of Diagnostic, Interventional and Pediatric Radiology, Inselspital, University Hospital Bern, Bern, 3010, Switzerland; Magnetic Resonance Methodology Group, Institute of Diagnostic and Interventional Neuroradiology, Inselspital, University Hospital Bern, Bern, 3010, Switzerland; Department of Diagnostic, Interventional and Pediatric Radiology, Inselspital, University Hospital Bern, Bern, 3010, Switzerland; Department of Radiology and Nuclear Medicine, Lucerne Cantonal Hospital, University of Lucerne, Lucerne, 6000, Switzerland

**Keywords:** chronic liver disease, cirrhosis, diffusion, diffusion-weighted imaging, fibrosis, intravoxel incoherent motion imaging, magnetic resonance elastography, MRI, perfusion, portal hypertension

## Abstract

**Background:**

With chronic liver disease (CLD) rising globally, noninvasive methods are needed to stratify early, intermediate, and advanced CLD with and without clinically significant portal hypertension (CSPH).

**Purpose:**

To analyze the combined diagnostic value of intravoxel incoherent motion (IVIM) and liver stiffness (LS) from magnetic resonance elastography (MRE) for CLD stage discrimination vs secondary single-parameter analyses.

**Materials and methods:**

This retrospective cross-sectional study included 185 patients who underwent 3T liver MRI, including MRE and IVIM, between March 2016 and November 2023. Patients with CLD were grouped based on their liver fibrosis degree into early CLD (F0-F1; *n* = 21), intermediate CLD (F2; *n* = 19), advanced CLD (F3-F4, *n* = 20), and advanced CLD with CSPH (*n* = 22). CSPH was defined as splenomegaly (>120 mm) with thrombocytopenia (<100 × 10^9^/L), ascites, or portosystemic collaterals. Patients without CLD (*n* = 103) served as negative controls. IVIM parameters (tissue diffusivity D, perfusion fraction f, and pseudo-diffusion coefficient D*) and MRE LS were analyzed. Statistical analysis included the Kruskal-Wallis test and both univariate and multivariate regression.

**Results:**

In total, 185 patients (median age: 55 years, interquartile range 25%-75%: 45-63 years; 94 men) were evaluated. All parameters differed significantly between all groups (*P* < .001). f and D* decreased with disease progression, while LS increased. D initially decreased in patients with CLD but increased in those with CSPH. Consequently, higher D-values indicated the presence of CSPH in advanced stages (odds ratio [OR] 1.09, 95% CI 1.03-1.17, *P* = .009). Elevated LS values showed strong associations with the presence of CLD (OR 5.01, CI 2.25-12.65, *P* < .001). Combining D and LS further improved diagnostic differentiation between disease stages, especially for differentiation between advanced CLD and advanced CLD with CSPH (OR 2.41, CI 1.33-5.44, *P* = .01).

**Conclusion:**

IVIM and MRE are useful for characterizing CLD and CSPH. Combining D from IVIM with LS from MRE improves diagnostic accuracy compared to MRE alone.

Abbreviations CI = 95% confidence interval; CLD = Chronic liver disease; CSPH = Clinically significant portal hypertension; D = Tissue diffusivity; D* = Pseudo-diffusion coefficient; IVIM = Intravoxel incoherent motion; LS = Liver stiffness; MRE = Magnetic resonance elastography; OR = Odds ratio; f = Perfusion fraction

**Summary** 

Combining intravoxel incoherent motion imaging (IVIM) with magnetic resonance elastography (MRE) improves noninvasive stratification of chronic liver disease and clinically significant portal hypertension (CSPH) compared with MRE alone.

**Key Results** 

- Liver perfusion fraction and pseudo-diffusion coefficient on intravoxel incoherent motion (IVIM) MRI decrease with disease progression, while liver stiffness (LS) on MR elastography increases.
- Tissue diffusivity (D) decreases in early CLD but is higher in advanced CLD with clinically significant portal hypertension (CSPH).
- Combining D from IVIM and LS from MRE improved differentiation between disease stages, enhancing noninvasive detection of CLD and CSPH.

## Introduction

Chronic liver disease (CLD) is characterized by persistent liver injury leading to inflammation, fibrosis, and ultimately cirrhosis. CLD is associated with significant morbidity and mortality due to the risk of hepatocellular carcinoma and life-threatening complications from clinically significant portal hypertension (CSPH, defined as hepatovenous pressure gradient ≥10 mmHg),^1^ such as variceal hemorrhage, hepatorenal syndrome, and spontaneous bacterial peritonitis.^2–5^

Annually, CLD accounts for approximately 2 million deaths worldwide, representing about 4% of all deaths.^6^ Chronic hepatitis C remains the leading cause of cirrhosis-related deaths globally, followed by alcohol-related liver disease.^7^ However, over the past decade, the burden of cirrhosis due to metabolic dysfunction-associated steatotic liver disease has increased markedly.^7^ Metabolic dysfunction-associated steatotic liver disease now affects about a quarter of the global adult population and is the second most common cause of cirrhosis in Europe and the United States after alcohol consumption.^6^ With rising metabolic risk factors and an aging population, the incidence of metabolic dysfunction-associated steatotic liver disease is expected to increase further.^6^ Given the growing prevalence of CLD worldwide,^7^ there is a need for accessible, noninvasive methods to detect CLD and CSPH in clinical practice.

Liver stiffness (LS) measurement by magnetic resonance elastography (MRE) is an MRI technique comparable to ultrasound-based methods such as FibroScan^®^, commonly used to assess liver fibrosis in CLD patients.^8^ MRE offers advantages, including whole-liver coverage, independence from obesity, and superior accuracy in grading fibrosis compared to FibroScan^®^.^9–12^

Intravoxel incoherent motion (IVIM) imaging is a diffusion-weighted MRI technique that quantifies the translational particle movements within tissue.^13^ Unlike conventional diffusion-weighted imaging, IVIM uses multiple *b* to distinguish between microperfusion in the capillaries and Brownian motion in the interstitial space.^13^ IVIM parameters include perfusion fraction (f), tissue diffusivity (D), and pseudo-diffusion coefficient (D*), whereby f reflects microcirculation in tissue diffusion, D reflects pure molecular diffusion (slow component), and D* represents perfusion-related diffusion (fast component).^14^^,^^15^

Since CLD and CSPH lead to significant changes in liver microarchitecture and microperfusion, we hypothesized that combining LS measurements from MRE with IVIM diffusion-weighted imaging parameters could improve noninvasive characterization of patients with CLD and CSPH.

The primary aim of this study was to evaluate the discriminatory value of combining LS with IVIM parameters (f + LS, D + LS, D* + LS) for differentiating CLD stages (early, intermediate, advanced, and advanced with CSPH), with a secondary aim to compare these combinations with the individual discriminatory value of LS and each IVIM parameter across these stages.

## Materials and methods

### Study sample

This retrospective cross-sectional study was approved by the regional ethics committee (Cantonal Ethics Committee, Canton of Bern, Switzerland). All patients gave written informed consent. Inclusion criteria were liver MRI with MRE and IVIM performed between March 2016 and November 2023 at the University Hospital of Bern (Switzerland). Exclusion criteria included inadequate IVIM quality (defined as resolution inadequate to clearly identify intrahepatic vessels, the gallbladder, and the liver contour or presence of extensive image artefacts) or inadequate MRE quality (defined as a small or absent region of high confidence or extensive image artefacts), portal vein thrombosis, liver iron overload, prior liver transplantation, and hemihepatectomy. Patients without a clinical history of CLD, who did not undergo liver biopsy, served as controls (no CLD).

### Imaging techniques

Examinations were performed on 3 Tesla MR scanners (Magnetom Verio/Magnetom Prisma; Siemens Healthineers, Erlangen, Germany).

Spin-echo-based MRE sequence (acquisition time 10 minutes, flip angle 90°, field of view 420 × 420 mm, matrix size 196 × 196, echo time 41 ms, repetition time 3200 ms) was conducted during a breath-hold using an MRE-driver (Resoundant, Inc., Rochester, United States) at 60 Hertz with 4 slices (5 mm thick, 20% interslice distance) in the middle of the liver, following Quantitative Imaging Biomarkers Alliance guidelines.^16^

For IVIM, a commercially available standard IVIM sequence (Siemens, Erlangen, Germany) with axial diffusion-weighted images (acquisition time 5 minutes, flip angle 90°, field of view 338 × 268 mm, matrix size 216 × 268, repetition time 2000 ms) was acquired at *b* values of 0, 10, 20, 50, 100, 180, 300, 420, 550, and 700 (s/mm^2^). *b* values were selected based on previous experience and tested in several volunteer examinations prior to the start of the study. For each *b* value, the number of signal averages was 6 (except for *b* = 0 s/mm^2^ with only one average). Seven respiratory-triggered axial slices (5 mm thick, 7.5 mm spacing) were collected per *b* value to cover the liver. An echo time of 53.2 ms was chosen to reduce distortions and avoid overestimation of the perfusion fraction in IVIM analysis.^17^

### Image analysis

IVIM sequence was analyzed by 2 residents in training (4 and 2 years of liver MRI experience, D.C. and S.U.vD.), both blinded to clinical information. Patients were randomly assigned to each resident. IVIM evaluation used an in-house interactive-data-language program (version 6.3, Research Systems Inc., Boulder, Colorado, United States). For cases requiring further clarification, image evaluation was performed in consensus with a second radiologist (A.T.H., 14 years of experience in liver MRI).

**Figure 1.**
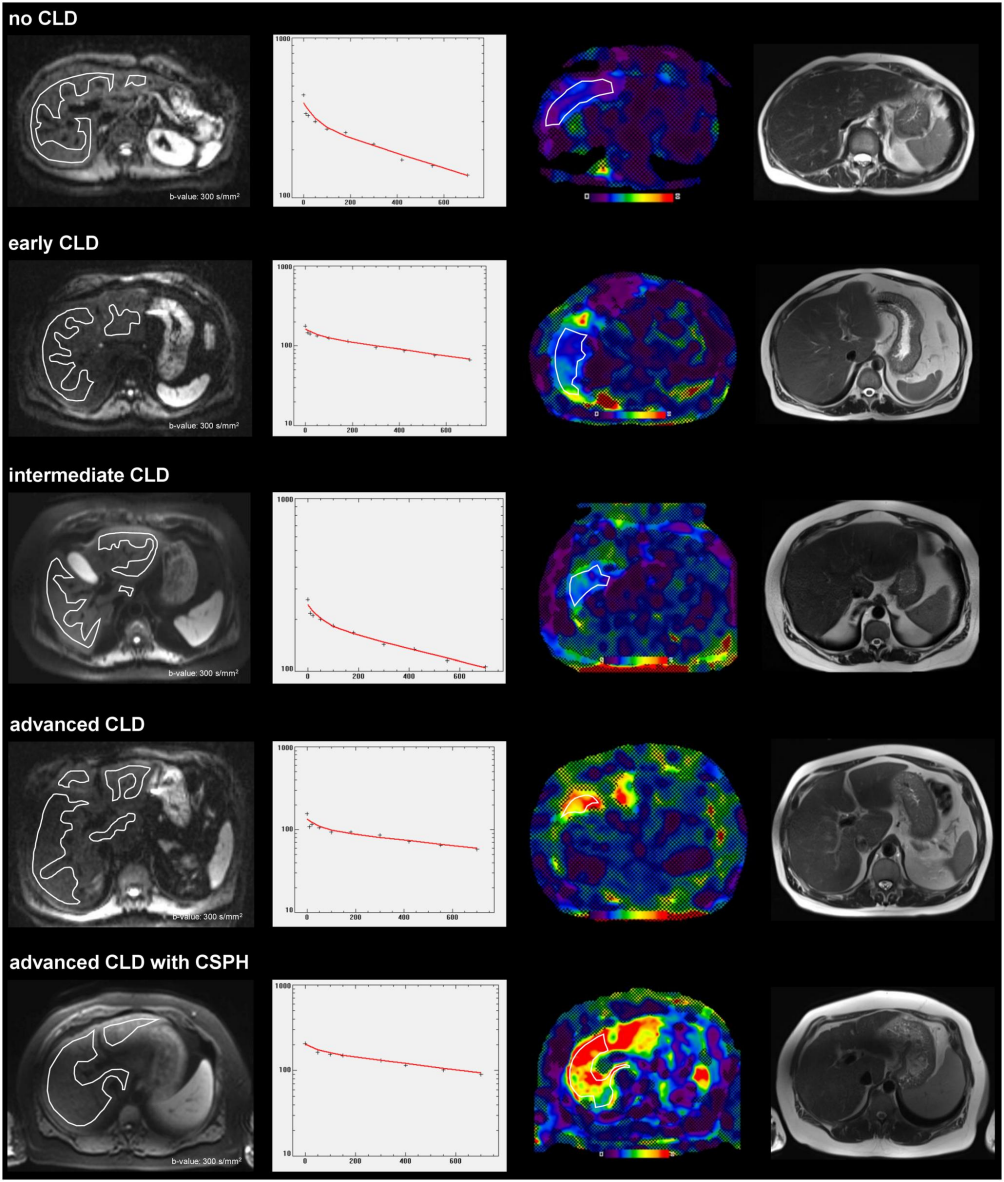
Measurement of IVIM parameters and liver stiffness in different patient groups. IVIM and MRE measurements across all 5 groups. The first column displays axial slices from the IVIM sequence at a *b* value of 300 s/cm^2^ with region(s) of interest. The second column shows fitting curves provided by the IVIM-measurement software. The third column shows slices from the MRE sequence with the region of interest drawn in the right liver lobe. Note that regions outside the 95% CI are automatically shaded in the MRE sequence. The right column depicts axial images acquired using a T2-HASTE sequence. ***no CLD*:** Image examples of a 60-year-old female patient. This patient showed an f of 27%, a D* of 666 × 10^−5^ mm^2^/seconds, a D of 121 × 10^−5^ mm^2^/seconds, and a liver stiffness of 1.8 kilopascal. ***early CLD*:** Image examples of a 47-year-old male patient. This patient showed an f of 23%, a D* of 552 × 10^−5^ mm^2^/seconds, a D of 114 × 10^−5^ mm^2^/seconds, and a liver stiffness of 3.5 kilopascal. ***intermediate CLD*:** Image examples of a 59-year-old male patient. This patient showed an f of 23%, a D* of 563 × 10^−5^ mm^2^/seconds, a D of 111 × 10^−5^ mm^2^/seconds, and a liver stiffness of 3.2 kilopascal. ***advanced CLD*:** Image examples of a 57-year-old male patient. This patient showed an f of 20%, a D* of 439 × 10^−5^ mm^2^/seconds, a D of 102 × 10^−5^ mm^2^/seconds, and a liver stiffness of 4.1 kilopascal. ***advanced CLD with CSPH*:** Image examples of a 54-year-old male patient. This patient showed an f of 15%, a D* of 426 × 10^−5^ mm^2^/seconds, a D of 92 × 10^−5^ mm^2^/seconds, and a liver stiffness of 7.2 kilopascal. Abbreviations: CLD = chronic liver disease; CSPH = clinically significant portal hypertension; D = tissue diffusivity; D* = pseudo-diffusion coefficient; f = perfusion fraction; IVIM = intravoxel incoherent motion; MRE = magnetic resonance elastography.

Regions of interest were placed in order to capture ≥50% of the liver parenchyma, while avoiding blood vessels, gallbladder, bile ducts, and keeping a 1 cm distance from the liver capsule (Figure 1). Mean liver values for each IVIM parameter were calculated from the individual regions of interest, adjusting for differences in region size. The following parameters were generated as described by Thoeny et al^18^: f, D, and D* by biexponential fitting of all *b* values. IVIM parameters are reported in units of (×10^−5^ mm^2^/seconds).

The MRE sequence for LS was independently assessed by 2 board-certified radiologists (14 and 10 years of liver MRI experience, A.T.H. and V.C.O.), both blinded to clinical information. Disagreements were resolved by consensus. Sectra Workstation PACS IDS7 (version 21.2, Sectra AB, Linköping, Sweden) was used for MRE assessment. Regions of interest were drawn as large as possible (≥500 pixels) within the right liver lobe on all 4 slices of the 95% confidence maps, maintaining a 1 cm distance from the liver capsule and avoiding vessels, gallbladder, bile ducts, and the area below the passive driver, according to Quantitative Imaging Biomarkers Alliance criteria^16^ (Figure 1). An average LS value (kilopascal) was calculated from the 4 slices, weighted by pixel count per region of interest.

All patients with CLD were screened for signs of CSPH on axial T2-weighted sequences, as well as non-contrast and contrast-enhanced axial T1 vibe Dixon sequences.

To assess interrater reliability, f, D*, D, and LS were independently measured by a second reader (S.U.vD., 2 years of liver MRI experience) in 30 randomly selected patients (15 with and 15 without CLD), blinded to clinical data and previous measurements.

### Clinical data

Clinical data collected within 3 months of the MRI examination included CLD etiology, sex, age, body mass index, history of arterial hypertension, smoking, regular alcohol consumption (defined as ≥2 alcoholic beverages per day for men and ≥1 alcoholic beverage for women or history of abusive alcohol consumption), and diabetes mellitus. Laboratory parameters included alanine aminotransferase, aspartate aminotransferase, alkaline phosphatase, albumin, bilirubin, gamma-glutamyltransferase, thrombocyte count, and Quick-value. Patients with missing values for specific laboratory parameters were excluded only from the corresponding statistical analyses.

### Liver histopathology

All CLD patients had undergone an ultrasound-guided percutaneous or fluoroscopy-guided transjugular liver biopsy at inclusion. Specimens of liver biopsies were subjected to standardized histopathological processing at our hospital and were scored using the meta-analysis of histological data in viral hepatitis score (no fibrosis F0, portal tract fibrosis without septa F1, portal tract fibrosis with rare septa formation F2, portal tract fibrosis with numerous septa F3, bridging fibrosis/cirrhosis F4)^19^ by specialized pathologists.

### Disease severity classification

CLD patients were categorized by fibrosis stage: early CLD (F0-F1), intermediate CLD (F2), advanced CLD (F3-F4), and advanced CLD with CSPH, where CSPH was defined as splenomegaly (>120 mm) with thrombocytopenia (<100 × 10^9^/L), ascites, or portosystemic collaterals (recanalized umbilical vein, splenorenal or gastroesophageal varices).^20^

### Statistical analysis

Statistical analyses were conducted using GraphPad Prism (version 10.0.2, GraphPad Software, San Diego, California, United States) and IBM SPSS Statistics (version 25.0, IBM Corporation, Armonk, New York, United States) by D.C. All analyses used nonparametric tests, predefined before the statistical evaluation, without introducing new tests after data review. Statistical analysis included descriptive statistics with group comparisons using the Kruskal-Wallis test and Dunn’s post hoc test for continuous variables, or the chi-square test with a *Z*-test and Bonferroni correction for categorical data.^21^ In addition, univariate regression analysis for single parameters (f, D*, D, LS) was performed to assess discrimination between “no CLD vs early CLD,” “early CLD + intermediate CLD vs advanced CLD + advanced CLD with CSPH,” and “advanced CLD vs advanced CLD with CSPH.” To control for multiple testing in the univariate analyses, *P*-values were additionally adjusted using the Bonferroni method. Furthermore, multivariate regression analyses for parameter combinations (D + LS, D* + LS, f + LS) were performed to assess discrimination between “no CLD vs early CLD,” “early CLD + intermediate CLD vs advanced CLD + advanced CLD with CSPH,” and “advanced CLD vs advanced CLD with CSPH.” Lastly, intraclass correlation coefficients for interreader agreement were calculated using a 2-way mixed effect model with absolute agreement.^22^ Here, values <0.5 indicated poor reliability, 0.5 to 0.75 moderate, 0.75 to 0.9 good, and >0.9 excellent reliability.^22^ In addition, Bland-Altman plots were generated to graphically represent the interrater variation. For all statistical analyses, a *P*-value of ≤.05 was considered significant.

## Results

### Patient characteristics

**Figure 2.**
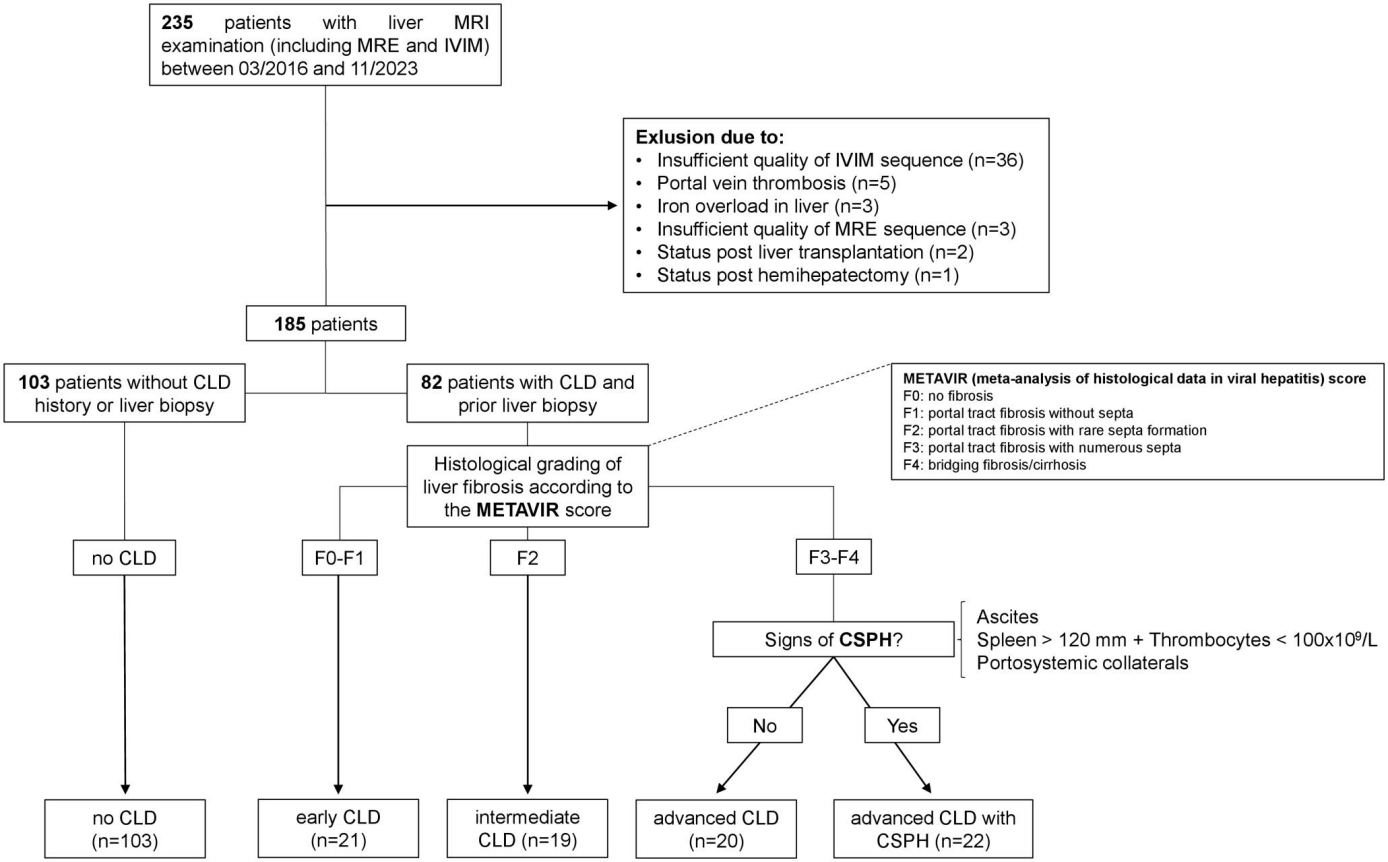
Flowchart of study sample. Abbreviations: CLD = chronic liver disease; CSPH = clinically significant portal hypertension; IVIM = intravoxel incoherent motion; MRE = magnetic resonance elastography.

From the initial 235 patients, 50 were excluded due to the following reasons: insufficient quality of IVIM sequences (*n* = 36), portal vein thrombosis (*n* = 5), iron overload in liver (*n* = 3), insufficient quality of MRE sequences (*n* = 3), status post liver transplantation (*n* = 2) and status post hemihepatectomy (*n* = 1). Examples of patients excluded due to insufficient image quality are attached as Figure S1. Of the finally included 185 patients (median age 55 years, 25%-75%: 45-63 years; 94 men), 103 had no CLD and 82 had biopsy-proven CLD (Figure 2). Among the patients with CLD, 4 had ascites at the time of the MR examination, and 9 had a history of prior hepatic decompensation. Patients with CLD were further subdivided into early CLD (*n* = 21), intermediate CLD (*n* = 19), advanced CLD (*n* = 20), and advanced CLD with CSPH (*n* = 22). Reasons for CSPH assignment are attached as Figure S2. Patient characteristics are displayed in Table 1. There was no evidence of age or sex differences between groups. Patients with CLD had a higher body mass index and a higher prevalence of arterial hypertension, diabetes mellitus, regular alcohol consumption, and smoking. Aspartate aminotransferase, alanine aminotransferase, bilirubin, and gamma-glutamyltransferase levels were higher and thrombocyte levels lower in patients with CLD, especially in those with CSPH. In addition, patients with CLD showed lower Quick-values than healthy patients. Metabolic dysfunction-associated steatotic liver disease (44%) emerged as the most common cause of CLD in our study sample, followed by chronic viral hepatitis (20%), alcohol-related liver disease (18%), cholestatic liver disease (7%), and autoimmune hepatitis (6%).

**Table 1.** Patient characteristics.

Parameter	No CLD (*n* = 103)	Missing values (*n*, %)	Early CLD (*n* = 21)	Missing values (*n*, %)	Intermediate CLD (*n* = 19)	Missing values (*n*, %)	Advanced CLD (*n* = 20)	Missing values (*n*, %)	Advanced CLD with CSPH (*n* = 22)	Missing values (*n*, %)	*P*-value
**Male (*n*, %)**	43 (42)	0 (0)	14 (67)	0 (0)	11 (58)	0 (0)	12 (60)	0 (0)	14 (64)	0 (0)	.09
**Age (years)**	52 (42-61)	0 (0)	50 (44-62)	0 (0)	57 (48-61)	0 (0)	61 (43-68)	0 (0)	58 (51-65)	0 (0)	.30
**Body mass index (kg/m^2^)**	25 (22-28)	1 (1)	27 (25-32)	0 (0)	30 (23-33)	0 (0)	29 (25-32)	0 (0)	27 (23-31)	1 (5)	.02
**Arterial hypertension (*n*, %)**	20 (19)	0 (0)	10 (48)	0 (0)	10 (53)	0 (0)	9 (45)*^,^**^,^***	0 (0)	4 (18)*^,^**^,^***	0 (0)	.002
**Smoking (*n*, %)**	15 (15)	0 (0)	7 (33)*	0 (0)	5 (26)*	0 (0)	3 (15)*	0 (0)	9 (41)**^,^***^,^****	0 (0)	.03
**Alcohol consumption (*n*, %)**	1 (1)	0 (0)	1 (5)	0 (0)	3 (16)*^,^**	0 (0)	2 (10)***	0 (0)	0 (0)***	0 (0)	.01
**Diabetes mellitus (*n*, %)**	6 (6)	0 (0)	2 (10)*	0 (0)	5 (26)*	0 (0)	8 (40)**^,^***	0 (0)	7 (32)**^,^***	0 (0)	<.001
**Alanine aminotransferase (U/L)**	22 (17-32)	43 (42)	69 (32-88)*	4 (19)	57 (34-131)*	0 (0)	44 (28-85)*	1 (5)	32 (25-46)	0 (0)	<.001
**Aspartate aminotransferase (U/L)**	22 (19-26)	56 (54)	36 (27-52)*	6 (29)	38 (32-88)*	0 (0)	50 (32-79)*	1 (5)	43 (32-64)*	1 (5)	<.001
**Alkaline phosphatase (U/L)**	72 (54-84)	58 (56)	78 (59-112)	4 (19)	80 (66-115)	0 (0)	103 (53-151)	1 (5)	94 (65-146)	0 (0)	.08
**Albumin (g/L)**	36 (35-39)	66 (64)	38 (34-41)	5 (24)	37 (35-40)	4 (21)	38 (33-42)	1 (5)	37 (32-38)	0 (0)	.64
**Bilirubin (µmol/L)**	7 (5-12)	61 (59)	8 (7-10)	4 (19)	10 (7-11)	1 (5)	9 (8-15)	1 (5)	17 (10-32)*^,^**	1 (5)	<.001
**Gamma-glutamyltransferase (U/L)**	21 (17-38)	50 (49)	57 (42-176)*	5 (24)	82 (50-153)*	0 (0)	76 (45-507)*	1 (5)	111 (66-225)*	0 (0)	<.001
**Thrombocytes (×10^9^/L)**	265 (204-348)	77 (75)	221 (181-264)	4 (19)	236 (170-266)	1 (5)	207 (176-276)	4 (20)	141 (79-259)*	3 (14)	.009
**Quick (%)**	100 (99-100)	52 (50)	99 (86-100)	9 (43)	98 (90-100)	7 (37)	90 (85-100)	5 (25)	71 (60-87)*^,^**^,^***	1 (5)	<.001

Results are presented as median and interquartile range (25%-75%). *P*-values were calculated using the Kruskal-Wallis test with Dunn’s multiple comparison post hoc test or χ^2^ test with a post hoc test consisting of a *Z*-test with Bonferroni correction as appropriate.

* *P* < .05 in post hoc test with no CLD.

** *P* < .05 in post hoc test with early CLD.

*** *P* < .05 in post hoc test with intermediate CLD.

**** *P* < .05 in post hoc test with advanced CLD. Regular alcohol consumption was defined as ≥2 alcoholic beverages per day for men and ≥1 alcoholic beverage per day for women or a history of abusive alcohol consumption.

Abbreviations: CLD = chronic liver disease; CSPH = clinically significant portal hypertension.

### Group comparison

**Figure 3.**
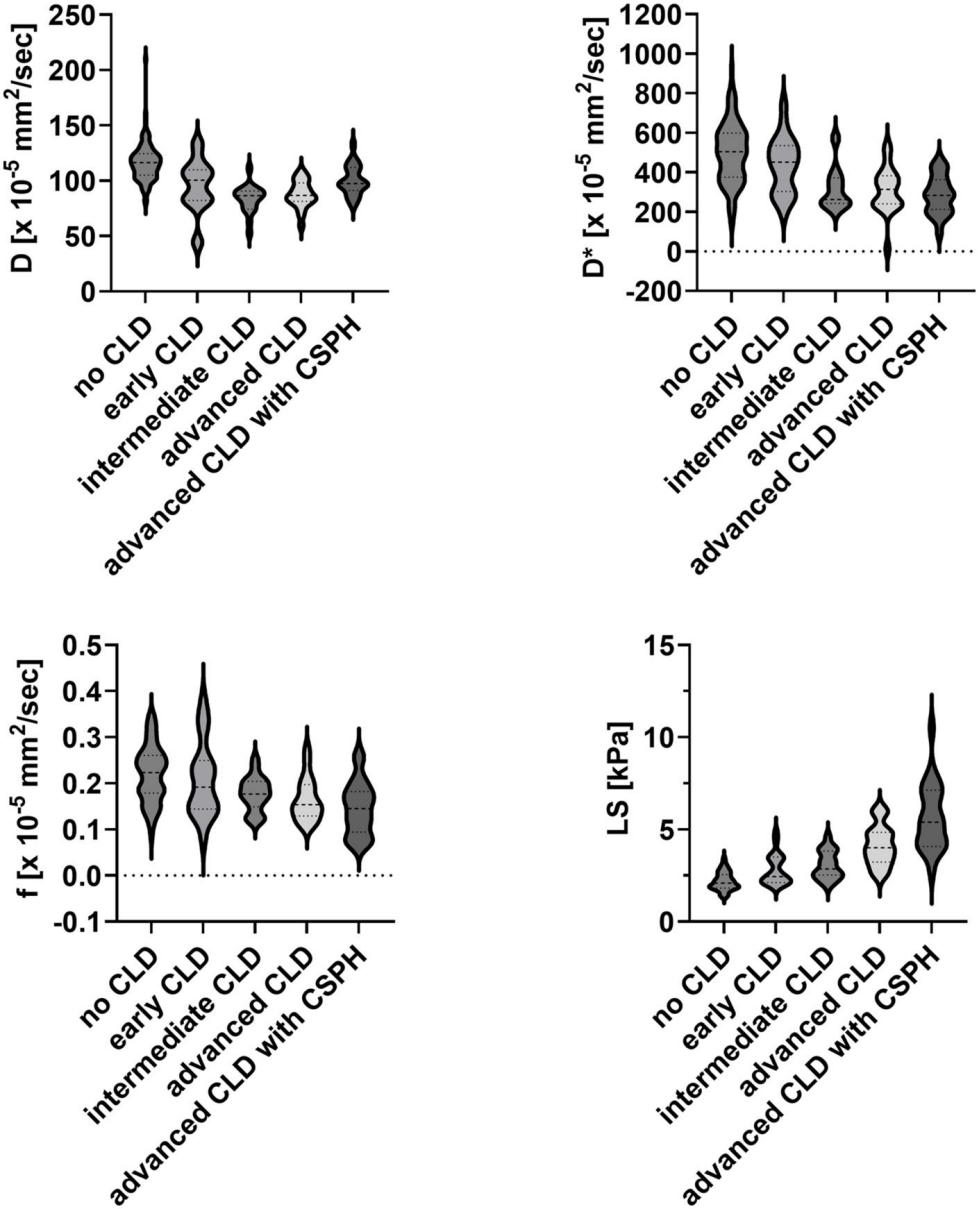
Violin plots of IVIM parameters and LS. Violin plots are presented for each subgroup for each parameter analyzed. Each violin plot contains 3 horizontal dashed lines: the top line marks the third quartile (Q3), the middle line marks the median (Q2), and the bottom line marks the first quartile (Q1). The width of the violin shape reflects the distribution of the data, with the broader sections indicating a higher concentration of values. The extent of the violin captures the full range of the observed data. Note that because the violin shape is based on kernel density estimation, the kernel density estimate can slightly extend into negative values, even though our dataset itself contains no negative observations. In the violin plots, it can be observed that f and D* values decline with disease progression, whereas LS values increase. The D parameter initially follows a similar downward trend as the other IVIM parameters in patients with CLD, but shifts in the opposite direction in patients with CSPH. Abbreviations: CLD = chronic liver disease; CSPH = clinically significant portal hypertension; D = tissue diffusivity; D* = pseudo-diffusion coefficient; f = perfusion fraction; IVIM = intravoxel incoherent motion; LS = liver stiffness.

All IVIM parameters (f, D*, D) and LS differed significantly between the different groups (Table 2, Figure 3). On closer inspection, one could notice that f and D* values decreased with disease progression, while LS values increased. Initially, D showed similar tendencies as the other IVIM parameters and decreased in patients with CLD, but interestingly increased in patients with CSPH, showing significantly higher D than those without CSPH. While D and LS showed a good discriminatory value between patients with and without CLD, D* did not. However, D* showed significant differences between patients with early and advanced CLD, notably in patients with CSPH.

**Table 2.** Comparison of IVIM parameters and liver stiffness between the groups.

Parameter	No CLD (*n* = 103)	Early CLD (*n* = 21)	Intermediate CLD (*n* = 19)	Advanced CLD (*n* = 20)	Advanced CLD with CSPH (*n* = 22)	*P*-value
**Tissue diffusivity (D) (×10** ^−^ **^5^ mm^2^/seconds)**	116 (105-124)	100 (82-110)*	87 (74-91)*	87 (81-98)*	97 (91-112)*	<.001
Pseudo-diffusion coefficient (D*) (×10 ^−^ **^5^ mm^2^/seconds)**	504 (376-598)	451 (304-536)	262 (243-372)*	314 (241-383)*	284 (212-362)*^,^**	<.001
**Perfusion fraction (f) (%)**	22 (18-26)	19 (14-25)	18 (15-20)	15 (13-20)*	15 (9-18)*	<.001
**Liver stiffness (LS) (kPa)**	2.1 (1.8-2.6)	2.4 (2.1-3.5)*	2.9 (2.5-3.8)*	4.0 (3.2-4.8)*^,^**	5.4 (4.1-7.1)*^,^**^,^***	<.001

Results are presented as median and interquartile range (25%-75%). *P*-values were calculated using the Kruskal-Wallis test with Dunn’s multiple comparison post hoc test or χ^2^ test with a post hoc test consisting of a *Z*-test with Bonferroni correction as appropriate.

* *P* < .05 in post hoc test with no CLD.

** *P* < .05 in post hoc test with early CLD.

*** *P* < .05 in post hoc test with intermediate CLD.

Abbreviations: CLD = chronic liver disease; CSPH = clinically significant portal hypertension; IVIM = intravoxel incoherent motion.

### Single parameters

Results of univariate regression analysis are shown in Table 3. Corresponding receiver operating characteristic curves are attached as Figure S3. In line with the results of the descriptive statistics, D was found to be helpful in differentiating no CLD vs early CLD and advanced CLD vs advanced CLD with CSPH but was less meaningful in the intermediate stages (early CLD + intermediate CLD vs advanced CLD vs advanced CLD with CSPH). While a higher D value in the early stages indicated the absence of CLD (odds ratio [OR] 0.93, CI 0.90-0.96, *P* < .001), a higher D value in the advanced stages indicated the presence of CSPH (OR 1.09, CI 1.03-1.17, *P* = .009).

**Table 3.** Results of univariate regression analysis.

Parameter	AUC	CI AUC	*P*-value AUC	OR	CI OR	*P*-value OR	Bonferroni-adjusted *P*-values
**No CLD vs early CLD**							
Tissue diffusivity (D)	0.77	0.64-0.89	<.001	0.93	0.90-0.96	<.001	.004
Pseudo-diffusion coefficient (D*)	0.62	0.49-0.75	.08	1.00	0.99-1.00	.09	.351
Perfusion fraction (f)	0.58	0.43-0.73	.24	0.97	0.90-1.05	.44	1.0
Liver stiffness (LS)	0.75	0.64-0.85	<.001	5.01	2.25-12.65	<.001	<.001
**Early CLD + intermediate CLD vs advanced CLD + advanced CLD with CSPH**							
Tissue diffusivity (D)	0.58	0.45-0.70	.22	1.02	0.99-1.04	.26	1.0
Pseudo-diffusion coefficient (D*)	0.66	0.54-0.78	.01	0.99	0.99-1.00	.006	.024
Perfusion fraction (f)	0.67	0.56-0.79	.007	0.89	0.81-0.96	.007	.029
Liver stiffness (LS)	0.89	0.82-0.96	<.001	4.94	2.66-10.96	<.001	<.001
**Advanced CLD vs advanced CLD with CSPH**							
Tissue diffusivity (D)	0.75	0.60-0.90	.006	1.09	1.03-1.17	.009	.036
Pseudo-diffusion coefficient (D*)	0.58	0.40-0.76	.38	1.00	0.99-1.00	.47	1.0
Perfusion fraction (f)	0.60	0.43-0.77	.27	0.93	0.82-1.05	.24	.956
Liver stiffness (LS)	0.76	0.61-0.90	.004	2.24	1.34-4.40	.007	.027

Abbreviations: AUC = area under the curve; CLD = chronic liver disease; CSPH = clinically significant portal hypertension; OR = odds ratio.

In contrast to D, D* and f were found to be particularly helpful in differentiating between early stages and advanced stages (early CLD + intermediate CLD vs advanced CLD + advanced CLD with CSPH). Here, increased D* and f values were associated with a lower probability of an advanced disease stage (OR 0.99, CI 0.99-1.00, *P* = .006; OR 0.89, CI 0.81-0.96, *P* = .007).

Finally, elevated LS levels were always significantly associated with an increased probability of early CLD (OR 5.01, CI 2.25-12.65, *P* < .001), advanced CLD (OR 4.94, CI 2.66-10.96, *P* < .001), and advanced CLD with CSPH (OR 2.24, CI 1.34-4.40, *P* = .007).

### Combined parameters

Results of multivariate regression analysis are shown in Table 4. Corresponding receiver operating characteristic curves are attached as Figure S4. Compared to the single parameters, higher areas under the curve could be achieved by combining IVIM parameters with LS. Of particular interest was the combination of D + LS, in which D (OR 1.10, 95% CI 1.03-1.19; *P* = .03) and LS (OR 2.41, 95% CI 1.33-5.44; *P* = .01) proved to be independent predictors in the differentiation of advanced CLD vs advanced CLD with CSPH. Furthermore, in this combination, D (OR 0.94, CI 0.90-0.97, *P* < .001) and LS (OR 4.26, CI 1.78-12.03, *P* = .003) proved to be independent predictors in the differentiation of no CLD vs early CLD.

**Table 4.** Results of multivariate regression analysis.

Parameter	AUC	CI AUC	*P*-value AUC	OR_1_	CI OR_1_	*P*-value OR_1_	OR_2_	CI OR_2_	*P*-value OR_2_
**No CLD vs early CLD**									
D + LS	0.84	0.74-0.94	<.001	0.94	0.90-0.97	<.001	4.26	1.78-12.03	.003
D* + LS	0.76	0.65-0.87	<.001	1.00	0.99-1.00	.09	5.35	2.30-14.31	<.001
f + LS	0.74	0.63-0.85	<.001	0.98	0.90-1.07	.68	4.99	2.23-12.69	<.001
**Early CLD + intermediate CLD vs advanced CLD + advanced CLD with CSPH**									
D + LS	0.89	0.82-0.95	<.001	1.00	0.96-1.03	.81	5.02	2.67-11.42	<.001
D* + LS	0.89	0.82-0.96	<.001	1.00	0.99-1.00	.22	4.64	2.49-10.33	<.001
f + LS	0.88	0.81-0.95	<.001	0.96	0.86-1.07	.49	4.67	2.48-10.49	<.001
**Advanced CLD vs advanced CLD with CSPH**									
D + LS	0.84	0.72-0.96	<.001	1.10	1.03-1.19	.03	2.41	1.33-5.44	.01
D* + LS	0.76	0.62-0.90	.004	1.00	0.99-1.01	.83	2.67	1.34-4.56	.008
f + LS	0.77	0.62-0.91	.003	0.98	0.85-1.13	.81	2.20	1.30-4.39	.01

Abbreviations: AUC = area under the curve; CLD = chronic liver disease; CSPH = clinically significant portal hypertension; D = tissue diffusivity; D* = pseudo-diffusion coefficient; f = perfusion fraction; LS = liver stiffness; OR_1_ = odds ratio of first parameter; OR_2_ = odds ratio of second parameter.

**Figure 4.**
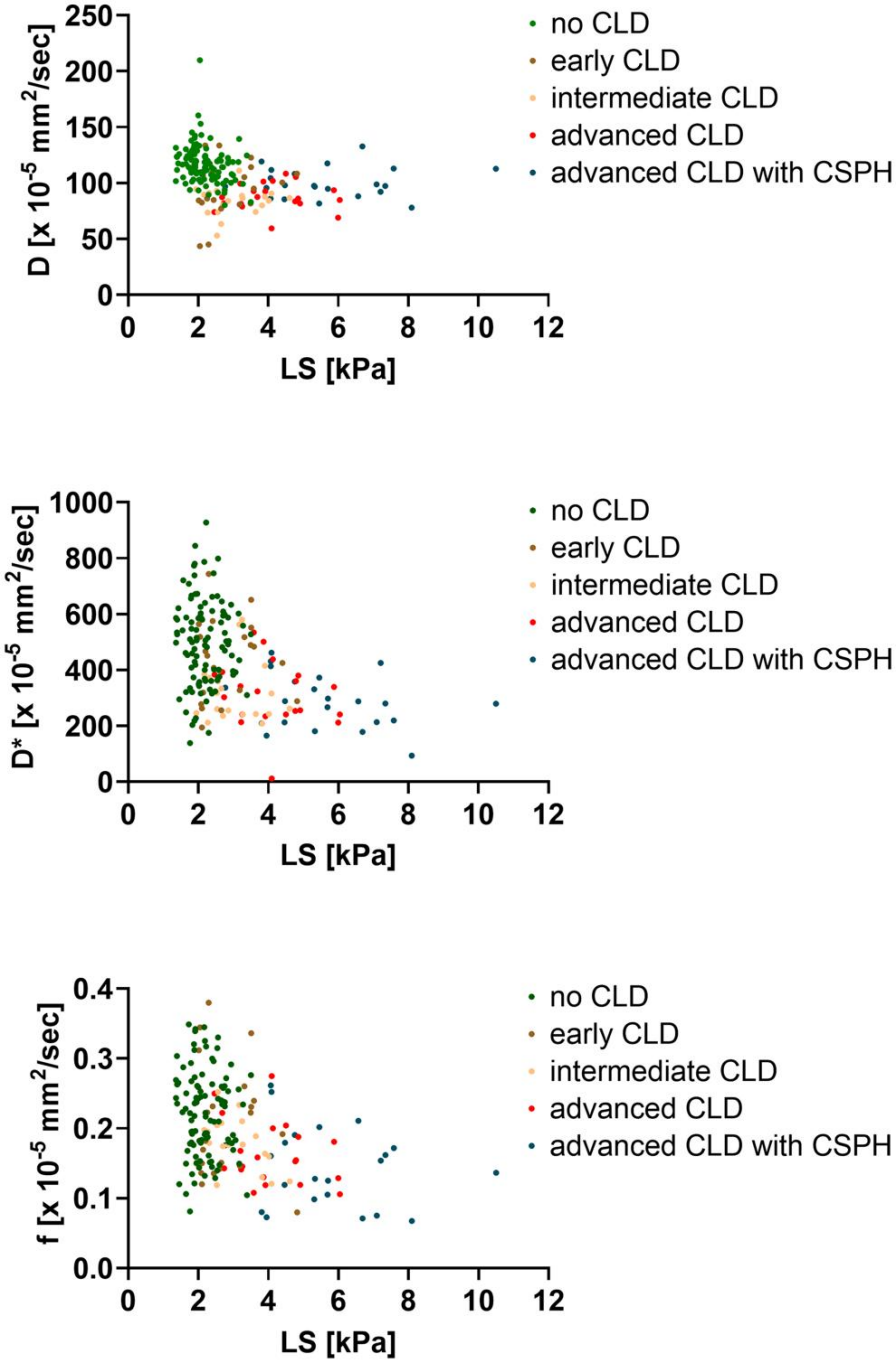
Scatter plots. Scatter plots with IVIM parameters on the *Y*-axis and LS on the *X*-axis are presented. Each point corresponds to a single patient, with the points colored differently depending on the group (green for no CLD, brown for early CLD, beige for intermediate CLD, red for advanced CLD, and blue for advanced CLD with CSPH). The scatter plots demonstrate a progressive increase in liver stiffness with advancing disease severity. Furthermore, increasing disease severity is generally associated with a decline in IVIM parameters (D, D*, f), although a subsequent rise in D is observed in patients with advanced CLD with CSPH. Abbreviations: CLD = chronic liver disease; CSPH = clinically significant portal hypertension; D = tissue diffusivity; D* = pseudo-diffusion coefficient; f = perfusion fraction; IVIM = intravoxel incoherent motion; LS = liver stiffness.

Figure 4 presents scatter plots with IVIM parameters on the *Y*-axis and LS on the *X*-axis.

### Interrater reliability

**Figure 5.**
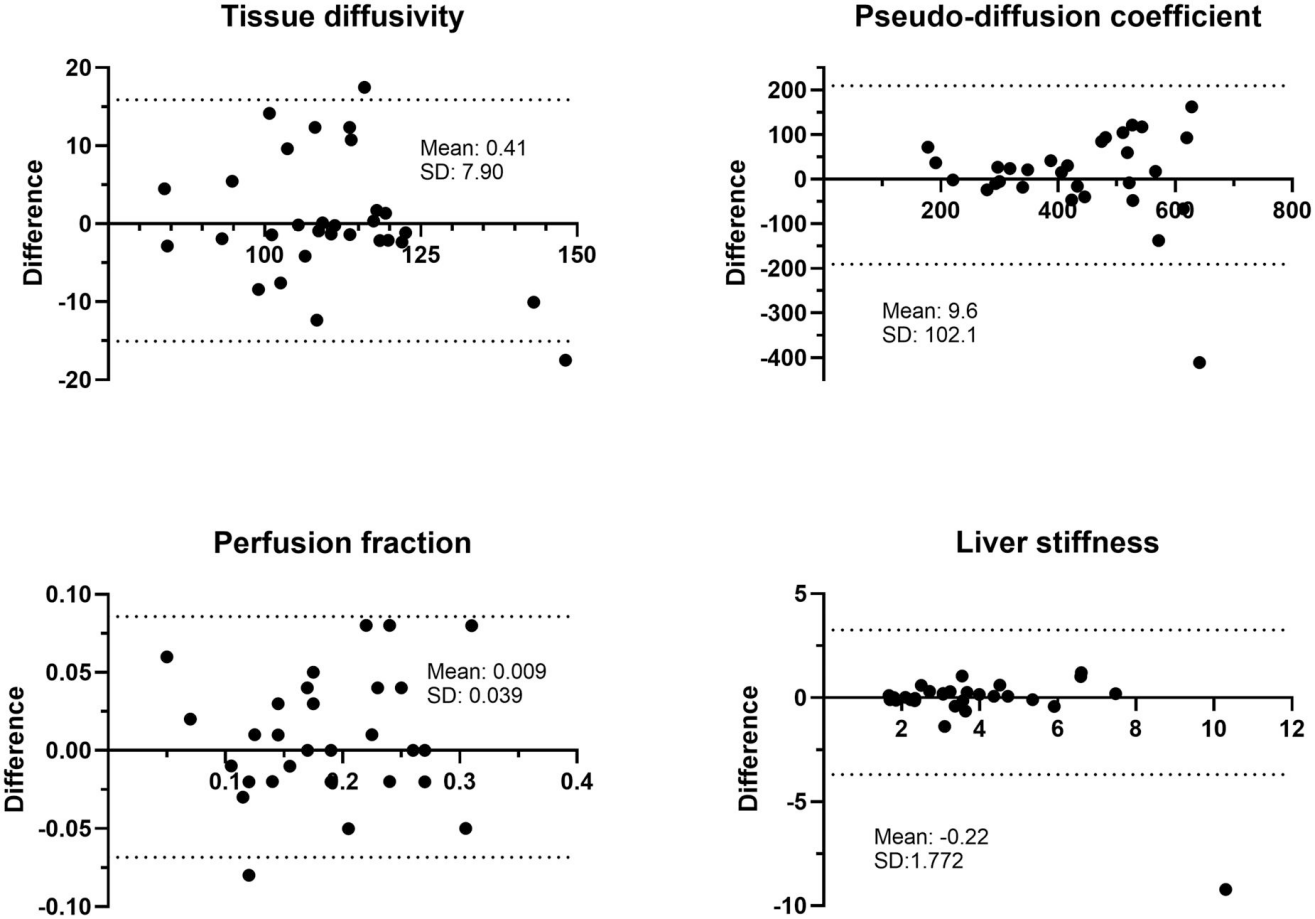
Bland-Altman plots for interrater comparison. Bland-Altman plots are shown to visualize the interrater comparison for the intravoxel incoherent motion (IVIM) parameters as well as for liver stiffness assessment. Each plot contains the mean difference of the measured values as well as the corresponding SD.

Excellent interrater reliability was observed for both D and f (both 0.92). Good interrater reliability was observed for D* and LS (0.86 and 0.81). Bland-Altman plots of interrater variation are shown in Figure 5.

## Discussion

This study shows that in patients with CLD, IVIM parameters of the liver (f, D*, D) and LS measured by MRE are associated with the severity of liver fibrosis and the presence of CSPH.

All IVIM parameters (f, D*, D) and LS differed significantly between the study groups. f and D* values decreased with disease progression, while LS values increased. Initially, D showed similar tendencies as the other IVIM parameters, but interestingly turned in the opposite direction in patients with CSPH, showing higher D than those without CSPH. In this context, it is known that the extracellular volume fraction of the liver increases with CLD progression due to collagen fiber deposition, restricting Brownian motion and therefore reducing diffusion with consequently lower values of D and D*.^23^^,^^24^ The increase of D in patients with CSPH may stem from fluid accumulation in the extracellular space, leading to facilitated Brownian motion, possibly caused by increased vascular permeability as demonstrated ex vivo in rat livers^25^ and in vivo in rabbit livers.^26^ However, this hypothesis needs further validation, including histological studies in human subjects.

Consequently, D was particularly useful in distinguishing between healthy patients and early stages of CLD, as well as between advanced stages with and without CSPH. Conversely, D* and f were more effective in differentiating early from advanced stages of CLD. Notably, LS consistently demonstrated a strong association with disease progression across all stages. The combination of IVIM parameters (especially D + LS) appeared to yield higher diagnostic accuracy than individual parameters alone, with D and LS emerging as potential independent predictors in both early and advanced disease stages. However, validation of our observations in future prospective human studies is required.

LS is known to be increased in liver fibrosis. In the past, MRE has been used to accurately grade liver fibrosis in different etiologies of CLD, including viral hepatitis,^27^ alcohol-related liver disease,^28^ and metabolic dysfunction-associated steatotic liver disease.^9^ In addition, LS assessed by MRE has been demonstrated to be elevated in the presence of CSPH,^29^ although CSPH has not yet been extensively studied in the context of MRE. As for the IVIM parameters, a recently published study by Ren et al, which examined IVIM parameters for fibrosis staging in patients with metabolic dysfunction-associated steatotic liver disease, obtained remarkably similar results to ours.^30^ Ren et al also noted that the parameters D* and f decreased with increasing fibrosis grade. In addition, Ren et al observed that D first decreases with increasing fibrosis grade before increasing again with further increase in fibrosis grade, as observed in our study.^30^ Finally, Ma et al have demonstrated in a porcine model that a combination of MRE and IVIM can be used to assess the hepatic venous pressure gradient.^31^ Thereby, Ma et al similarly observed an increase in LS and a decrease in f in CSPH, which correlated significantly with the hepatic venous pressure gradient.^31^ A tabular overview of the main findings of the studies mentioned in this paragraph and our findings is attached as Figure S5.

This study has several limitations. First, it was carried out at a single center using MRI scanners from a single vendor. For this reason, external studies to validate our preliminary findings are warranted—ideally, multicenter investigations with MRI systems from different vendors with liver biopsy serving as the reference standard. Another limitation of the present study is the high number of observed study exclusions due to poor IVIM quality, as IVIM quality is significantly degraded by motion artifacts. Unfortunately, the liver, particularly the left lobe, is especially prone to motion artifacts because of its proximity to the heart and the diaphragm.^32^ Development of faster IVIM sequences may mitigate these limitations in the future. In fact, several technical developments in IVIM have recently been introduced that accelerate image acquisition, reduce distortions, improve signal-to-noise ratio, and decrease motion sensitivity, thereby enabling more reliable IVIM parameter estimation.^33^^,^^34^ Nevertheless, in order to maintain methodological consistency over the course of this study, we refrained from integrating evolving sequence modifications, as doing so would have compromised the comparability of the data across the entire cohort. Finally, the diagnosis of CSPH in this study was based on MRI-detected morphological changes and laboratory parameters rather than direct invasive measurement of the hepatic venous pressure gradient, which may have introduced additional data heterogeneity. For this reason, future studies with direct comparisons of IVIM parameters and LS to the hepatovenous pressure gradient are needed in order to validate our results.

Other possible imaging techniques to be studied in the future for the assessment of CSPH include quantification of liver surface nodularity on MRI or CT, flow analysis of the hepatic and portosystemic vasculature using 4D flow MRI, MR relaxometry techniques such as liver T1 mapping, and measurement of splenic stiffness with MRE.^1^^,^^12^^,^^35^

In conclusion, both IVIM and MRE show promise for characterizing CLD and CSPH. While our findings suggest that combining D from IVIM with LS from MRE may provide complementary diagnostic information, further studies are warranted to confirm whether such integration consistently improves diagnostic accuracy over MRE alone.

## Supplementary Material

umaf039_Supplementary_Data

## Data Availability

The data underlying this article cannot be shared publicly to protect the privacy of study participants. An anonymized version of the data will be made available upon reasonable request to the corresponding author.
