# Supplementary material for: Detection and severity stratification of chronic liver disease using magnetic resonance intravoxel incoherent motion and elastography
Source: Radiol Adv. 2025 Nov 14;2(6):umaf039. doi: 10.1093/radadv/umaf039 (PMC12677949; doi:10.1093/radadv/umaf039)
Supplement: umaf039_Supplementary_Data [file umaf039_supplementary_data.zip › Supplementary_Material.zip!/Supplementary Material.pdf]

Detection and Severity Stratification of Chronic Liver Disease Using Magnetic Resonance Intravoxel Incoherent Motion and Elastography

Damiano Catucci (M.D.)[1,2]; Sandro Urs von Daeniken (M Med)[1]; Verena Carola Obmann (M.D.)[1,3]; Annalisa Berzigotti (M.D., Ph.D.)[4]; Lukas Ebner (M.D.)[1,5]; Johannes Thomas Heverhagen (M.D., Ph.D.)[1]; Andreas Christe (M.D.)[1]; Peter Vermathen (Ph.D.)[6]; Adrian Thomas Huber (M.D., Ph.D.)[1,5]

- [1]: Inselspital, University Hospital Bern, Department of Diagnostic Interventional and Pediatric Radiology, Bern, Bern, Switzerland
- [2]: Graduate School for Health Sciences, University of Bern, Bern, Bern, Switzerland
- [3]: Department of Radiology, Zuger Kantonsspital, 6340 Baar, Zug, Zug, Switzerland
- [4]: Inselspital, University Hospital Bern, Hepatology, Department of Visceral Surgery and Medicine, Bern, Bern, Switzerland
- [5]: Department of Radiology and Nuclear Medicine, Lucerne Cantonal Hospital, University of Lucerne, Luzern, Luzern, Switzerland
- [6]: Magnetic Resonance Methodology Group, Institute of Diagnostic and Interventional Neuroradiology, Inselspital, Bern University Hospital, University of Bern, Bern, Bern, Switzerland

Corresponding author

Damiano Catucci, M.D.

Department of Diagnostic, Interventional and Pediatric Radiology
Rosenbühlgasse 27, Inselspital
3010 Bern, Switzerland

damiano.catucci@insel.ch
Phone: +41 31 664 30 05

Figure S1

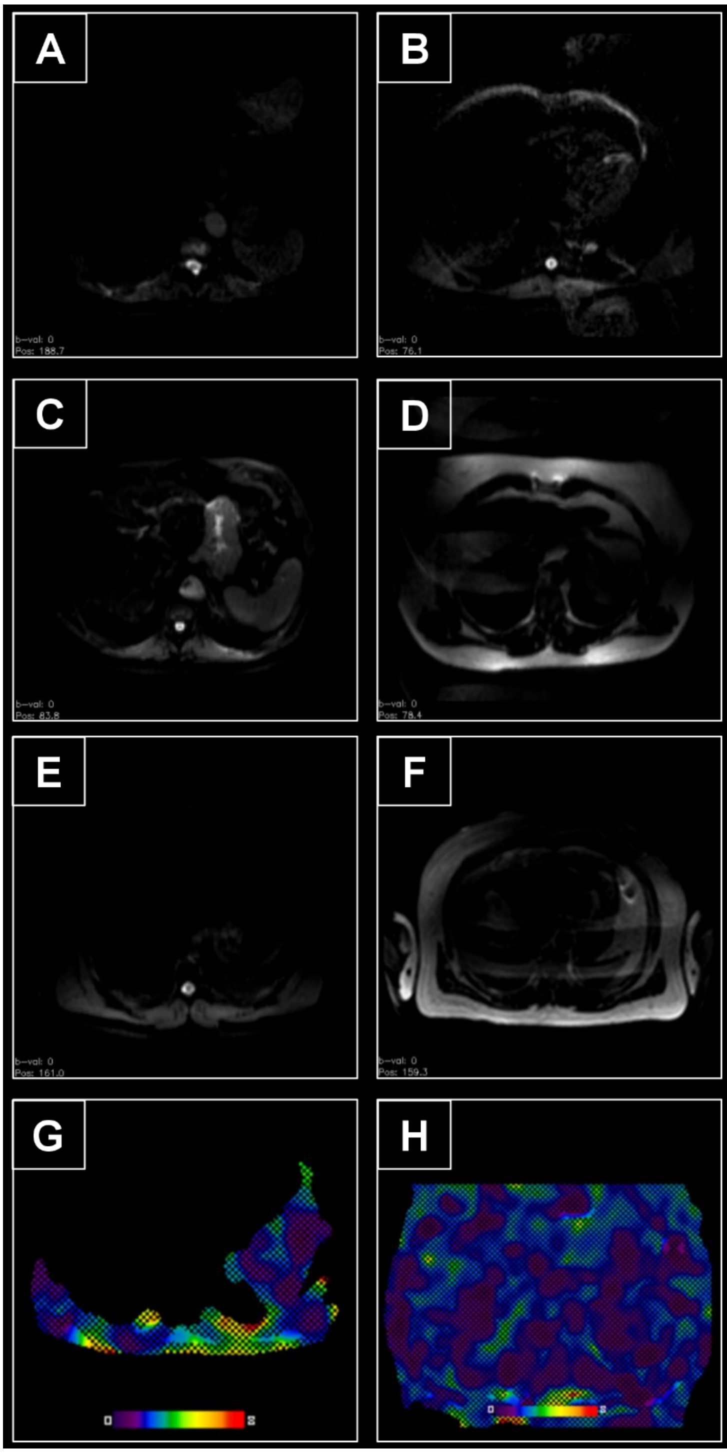

Images **A-F** are examples of patients who were excluded due to bad quality of intravoxel incoherent motion imaging sequence.

Images **G** and **H** are examples of patients who were excluded due to insufficient quality of magnetic resonance elastography.

Figure S2

Surrogate markers for clinically significant portal hypertension (CSPH) in patients with advanced chronic liver disease (CLD) were defined as:

- Splenomegaly (> 12 cm) with thrombocytopenia (< 100 x 10^9/L)
- Ascites
- Portosystemic collaterals (recanalized umbilical vein, splenorenal or gastroesophageal varices)

Exact reasons for assignment of patients with advanced CLD to the group “advanced CLD with CSPH” (n=22):

- Ascites without additional surrogate marker for CSPH (n=2)
- Splenomegaly with thrombocytopenia without additional surrogate marker for CSPH (n=1)
- Portosystemic collaterals with/without additional surrogate marker for CSPH (n=19)

Figure S3

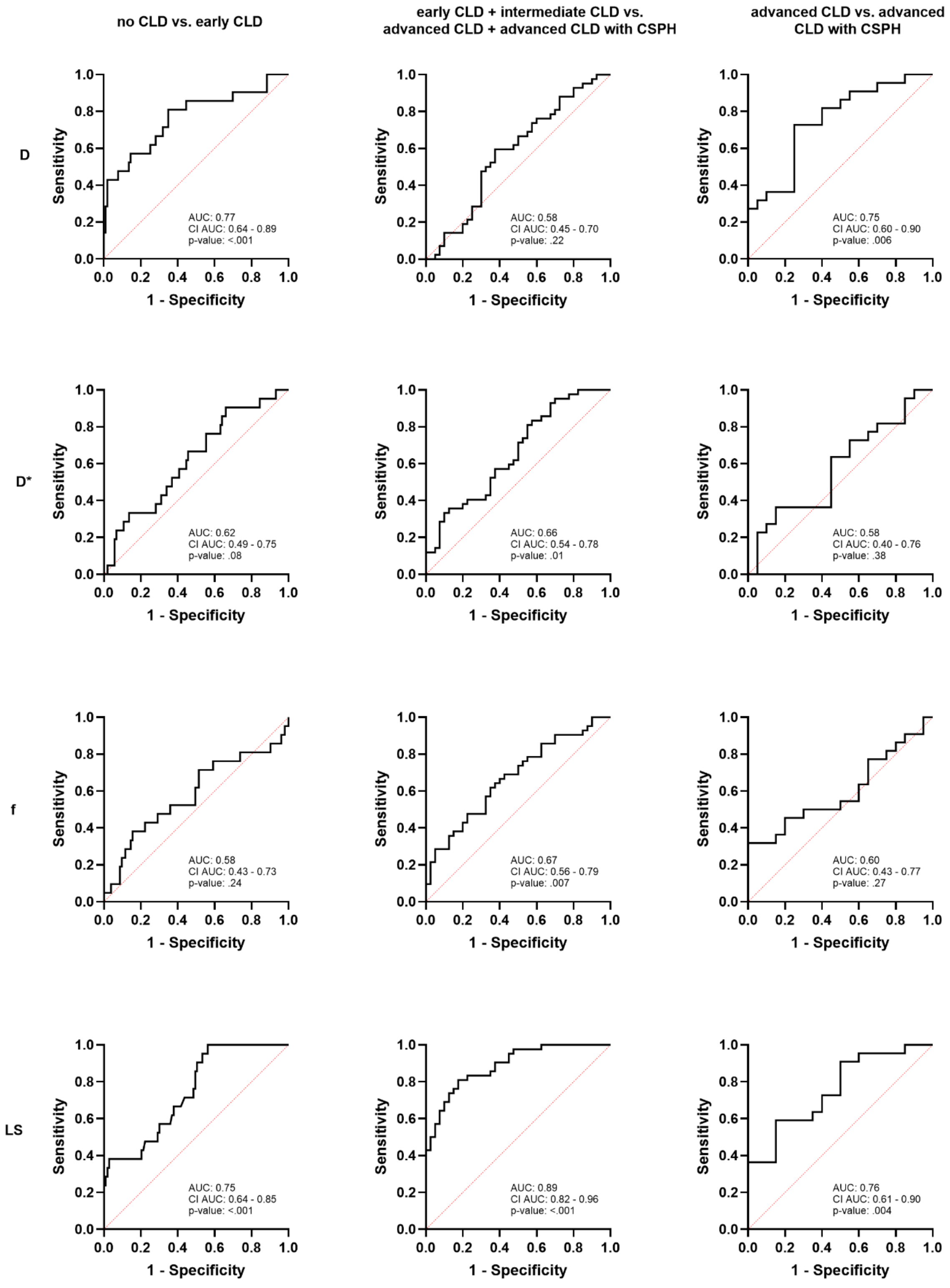

Receiver operating characteristics-curves of univeriate regression analysis

AUC = area under the curve; CI = 95% confidence interval; CLD = chronic liver disease; CSPH = clinically significant portal hypertension; D = tissue diffusivity; D = pseudo-diffusion coefficient; LS = liver stiffness; f = perfusion-fraction*

Figure S4

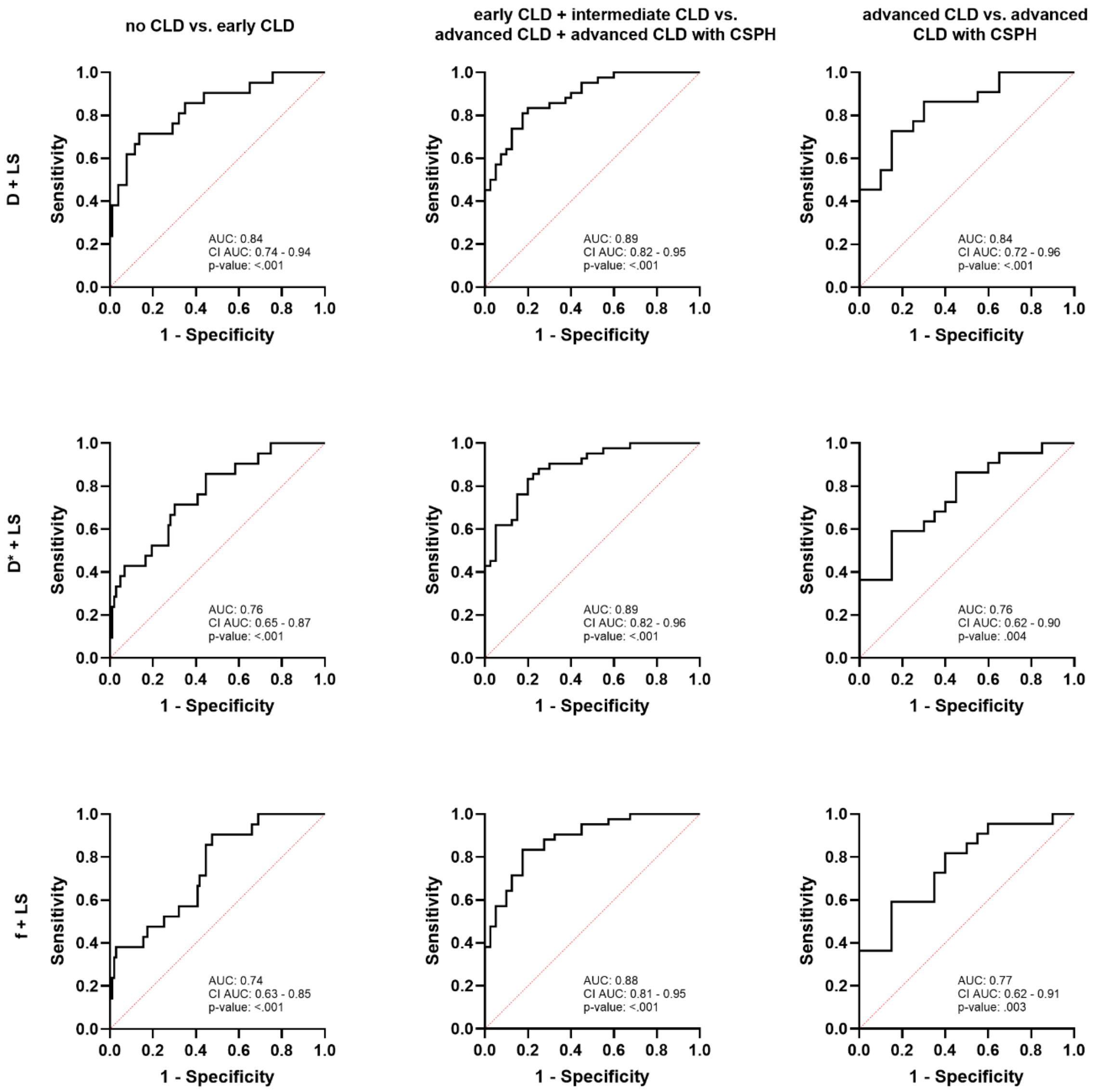

Receiver operating characteristics-curves of multivariate regression analysis

AUC = area under the curve; CI = 95% confidence interval; CLD = chronic liver disease; CSPH = clinically significant portal hypertension; D = tissue diffusivity; D = pseudo-diffusion coefficient; LS = liver stiffness; f = perfusion-fraction*

Figure S5

Study	LS	D	D*	f	Remarks
**Present study**	Increasing LS with increasing fibrosis and in patients with CSPH.<br><br>Median (IQR) no CLD: 2.1 (1.8-2.6) kPa<br>Median (IQR) early CLD: 2.4 (2.1-3.5) kPa<br>Median (IQR) intermediate CLD: 2.9 (2.5-3.8) kPa<br>Median (IQR) advanced CLD: 4.0 (3.2-4.8) kPa<br>Median (IQR) advanced CLD with CSPH: 5.4 (4.1-7.1) kPa	Initial decline with progression of CLD severity, followed by re-increase in CLD with CSPH.<br><br>Median (IQR) no CLD: 116 (105-124) (x 10^{-5} mm^2/sec)<br>Median (IQR) early CLD: 100 (82-110) (x 10^{-5} mm^2/sec)<br>Median (IQR) intermediate CLD: 87 (74-91) (x 10^{-5} mm^2/sec)<br>Median (IQR) advanced CLD: 87 (81-98) (x 10^{-5} mm^2/sec)<br>Median (IQR) advanced CLD with CSPH: 97 (91-112) (x 10^{-5} mm^2/sec)	Decrease in patients with liver fibrosis with and without CSPH compared to healthy patients.<br><br>Median (IQR) no CLD: 504 (376-598) (x 10^{-5} mm^2/sec)<br>Median (IQR) early CLD: 451 (304-536) (x 10^{-5} mm^2/sec)<br>Median (IQR) intermediate CLD: 262 (243-372) (x 10^{-5} mm^2/sec)<br>Median (IQR) advanced CLD: 314 (241-383) (x 10^{-5} mm^2/sec)<br>Median (IQR) advanced CLD with CSPH: 284 (212-362) (x 10^{-5} mm^2/sec)	Decrease in patients with liver fibrosis with and without CSPH compared to healthy patients.<br><br>Median (IQR) no CLD: 22 (18-26) %<br>Median (IQR) early CLD: 19 (14-25) %<br>Median (IQR) intermediate CLD: 18 (15-20) %<br>Median (IQR) advanced CLD: 15 (13-20) %<br>Median (IQR) advanced CLD with CSPH: 15 (9-18) %	None
**"Magnetic resonance elastography for staging liver fibrosis in non-alcoholic fatty liver disease: a diagnostic accuracy systematic review and individual participant data pooled analysis"**<br><br>**Singh et al., European Radiology, volume 26, 2016**	Increasing LS with increasing liver fibrosis stage.<br><br>Cutoff value F1: 2.88 kPa<br>Cutoff value F2: 3.54 kPa<br>Cutoff value F3: 3.77 kPa<br>Cutoff value F4: 4.09 kPa	n/a	n/a	n/a	No median or mean values for the groups were reported in this study.
**"Magnetic resonance elastography for the detection and staging of liver fibrosis in chronic hepatitis B"**<br><br>**Venkatesh et al., European Radiology, volume 24, 2014**	Increasing LS with increasing liver fibrosis stage.<br><br>Cutoff value F1: 2.74 kPa<br>Cutoff value F2: 3.2 kPa<br>Cutoff value F3: 3.7 kPa<br>Cutoff value F4: 4.33 kPa	n/a	n/a	n/a	No median or mean values for the groups were reported in this study.
**"Cutoff Values for Alcoholic Liver Fibrosis Using Magnetic Resonance Elastography Technique"**<br><br>**Bensamoun et al., Alcohol Clinical & Experimental Research, volume 37, 2013**	Increasing LS with increasing liver fibrosis stage.<br><br>Cutoff value F1: 2.20 kPa<br>Cutoff value F2: 2.57 kPa<br>Cutoff value F3: 3.31 kPa<br>Cutoff value F4: 4.4 kPa	n/a	n/a	n/a	No median or mean values for the groups were reported in this study.
**"Relation of Magnetic Resonance Elastography to Fontan Failure and Portal Hypertension"**<br><br>**Alsaied et al., American Journal of Cardiology, volume 124, 2019**	Increased LS in patients with signs of portal hypertension.<br><br>Mean ± SD patients without portal hypertension: 4.2 ± 0.8 kPa<br>Mean ± SD patients with Portal hypertension: 5.2 ± 1.3 kPa<br><br>Cutoff value portal hypertension: 4.5 kPa	n/a	n/a	n/a	This study examined patients with Fontan-circulation.
**"Intravoxel incoherent motion assessment of liver fibrosis staging in MASLD"**<br><br>**Ren et al., Abdominal Radiology, volume 49, 2024**	n/a	Initial decline with progression of fibrosis, followed by re-increase in severe fibrosis.<br><br>Mean ± SD F0: 0.623 ± 0.146 (x 10^{-3} mm^2/sec)<br>Mean ± SD F1: 0.551 ± 0.149 (x 10^{-3} mm^2/sec)<br>Mean ± SD F2: 0.584 ± 0.104 (x 10^{-3} mm^2/sec)<br>Mean ± SD F3: 0.602 ± 0.150 (x 10^{-3} mm^2/sec)<br>Mean ± SD F4: 0.707 ± 0.214 (x 10^{-3} mm^2/sec)	Decrease with progression of fibrosis.<br><br>Mean ± SD F0: 65.361 ± 10.605 (x 10^{-3} mm^2/sec)<br>Mean ± SD F1: 62.099 ± 8.433 (x 10^{-3} mm^2/sec)<br>Mean ± SD F2: 58.070 ± 8.724 (x 10^{-3} mm^2/sec)<br>Mean ± SD F3: 52.355 ± 7.462 (x 10^{-3} mm^2/sec)<br>Mean ± SD F4: 42.871 ± 8.422 (x 10^{-3} mm^2/sec)	Decrease with progression of fibrosis.<br><br>Mean ± SD F0: 0.413 ± 0.086<br>Mean ± SD F1: 0.393 ± 0.062<br>Mean ± SD F2: 0.345 ± 0.061<br>Mean ± SD F3: 0.312 ± 0.072<br>Mean ± SD F4: 0.291 ± 0.058	
**"Novel imaging-based approaches for predicting the hepatic venous pressure gradient in a porcine model of liver cirrhosis and portal hypertension"**<br><br>**Ma et al., Life Sciences, volume 264, 2021**	LS increased due to liver fibrosis induced following hepatic arterial embolization.<br><br>Mean ± SD 4 weeks after embolization: 3.51 ± 1.06 kPa<br>Mean ± SD 8 weeks after embolization: 4.22 ± 1.51 kPa<br>Mean ± SD 12 weeks after embolization: 4.86 ± 2.37 kPa	No significant changes were observed for D after embolization of the hepatic artery.	No significant changes were observed for D* after embolization of the hepatic artery.	f decreased after embolization of the hepatic artery and therefore in subjects with fibrosis.<br><br>Proposed model for estimation of the hepatovenous pressure gradient based on MRE and IVIM:<br><br>lg(hepatovenous pressure gradient) = 0.079 × LS-0.01 × f	All pigs included in the analysis reached F4 (cirrhosis) by week 8 after embolization. In addition, the mean value of the hepatovenous pressure gradient of the cohort reached 9.98 cmH_2O 4 weeks after embolization and remained high.<br><br>Mean values of D and D* were not reported explicitly in this study.

IVIM = intravoxel incoherent motion; CLD = chronic liver disease; CSPH = clinically significant portal hypertension; D = tissue diffusivity; D = pseudo-diffusion coefficient; LS = liver stiffness; f = perfusion fraction*
